# Parenteral Nutrition and Oxidant Load in Neonates

**DOI:** 10.3390/nu13082631

**Published:** 2021-07-30

**Authors:** Kandeepan Karthigesu, Robert F. Bertolo, Robert J. Brown

**Affiliations:** Department of Biochemistry, Memorial University of Newfoundland, St. John’s, NL A1B 3X9, Canada

**Keywords:** parenteral nutrition, preterm birth, oxidants, antioxidants, pro-oxidants, lipid peroxidation, parenteral nutrition-associated liver disease, gut atrophy

## Abstract

Neonates with preterm, gastrointestinal dysfunction and very low birth weights are often intolerant to oral feeding. In such infants, the provision of nutrients via parenteral nutrition (PN) becomes necessary for short-term survival, as well as long-term health. However, the elemental nutrients in PN can be a major source of oxidants due to interactions between nutrients, imbalances of anti- and pro-oxidants, and environmental conditions. Moreover, neonates fed PN are at greater risk of oxidative stress, not only from dietary sources, but also because of immature antioxidant defences. Various interventions can lower the oxidant load in PN, including the supplementation of PN with antioxidant vitamins, glutathione, additional arginine and additional cysteine; reduced levels of pro-oxidant nutrients such as iron; protection from light and oxygen; and proper storage temperature. This narrative review of published data provides insight to oxidant molecules generated in PN, nutrient sources of oxidants, and measures to minimize oxidant levels.

## 1. Introduction

Preterm or premature birth and its complications cause an enormous economic burden to a country. For instance, Canada spends nearly CAD 587.1 million for all premature infants [1]. In Canada, an estimated 8% of newborns are born prematurely at 37 weeks or less [2]. Moreover, over the last 14 years, the proportion of low-birth-weight babies (<2.5 kg) has increased from 5.9% in 2003 to 6.5% in 2017 [3]. Premature and low-birth-weight neonates may encounter difficulties such as respiratory, hepatocellular, and cardiovascular complications, and have a higher risk of developing chronic health conditions later in life [4]. The majority of complications in neonates are related to oxidative stress that develops early in life [5,6]. Newborns are at a higher risk of oxidative stress due to the exposure of oxidants at an early growing period, from various dietary sources [7]. Furthermore, if oxygen therapy is used during the neonatal period, this can be an additional source of oxidant load, exacerbating oxidative stress [8,9].

The gastrointestinal tract of premature neonates is immature and inefficient with respect to digestion, assimilation, and the absorption of nutrients for the newborn. Neonates depend on adequate early nutrition, which not only safeguards life, but also provides positive health outcomes in later life [10]. Starting feeds by mouth or nasogastric tube (enteral feeding) as quickly as possible stimulates the development and function of the gastrointestinal tract [11]. However, because of the lower gastrointestinal tract capacity, immature gut function or congenital problems, and high demand for nutrients for their accelerated growth, the consumption of recommended nutrients per day may require the intravenous administration of nutrients (i.e., parenteral nutrition (PN)), in addition to some enteral feeding. However, in some cases in extremely premature or low-birth-weight neonates, or neonates with congenital anomalies of the gastrointestinal tract, all nutrients might need to be delivered as total parenteral nutrition (TPN) [5]. PN is prepared by mixing elemental nutrients, including dextrose, amino acids, vitamins, minerals, and trace elements, and intravenously delivering a lipid emulsion infused either separately, or mixed with aqueous solutions.

Although TPN has become a crucial part of the clinical management of premature and newborn infants, it is subject to oxidation, and is thus a source of oxidant exposure to neonates [12]. Oxidants in TPN may also lead to the production of additional oxidation products in vivo, which may detrimentally affect the health of the infant. The oxidant load collectively generated in human neonates from various sources could lead to liver diseases, bronchopulmonary dysplasia (BPD), gut atrophy, necrotizing enterocolitis, and retinopathy [13,14]. Moreover, several animal experiments have demonstrated that the exogenous oxidized molecules from TPN could cause hepatocellular damage, cholestasis, apoptosis, and pulmonary fibrosis [15,16,17,18,19].

Despite in vivo antioxidants such as vitamin E, vitamin C, superoxide dismutase, catalase, and glutathione [20,21] playing a major role to reduce oxidative stress, neonates are even more prone to oxidative damage because antioxidant systems in newborns are immature, especially in preterm infants. Moreover, premature neonates, to whom TPN is prescribed more often, are more likely to be exposed to high amounts of peroxides from endogenous and exogenous sources. For instance, numerous endogenous sources of oxidative stress are from birth trauma, reperfusion injury from hypoxia, oxygen therapy, acidosis, phototherapy, mechanical ventilation, infection, and inflammation [22,23]. The exogenous sources of hydroperoxides are from oxidized TPN. The antioxidant systems within neonates that remove these peroxides work at a lower capacity. Indeed, neonates have lower amounts of vitamin E, β-carotene, and glutathione compared to adults [24,25,26]. Superoxide dismutase and catalase activities are also lower in preterm neonates compared to term neonates, children, and adults [27]. Although the antioxidant effects of the iron transport protein, apotransferrin, and the iron oxidizing protein, ceruloplasmin, are nearly 200-fold higher than that of vitamin E [28], they are found at lower concentrations in preterm than term neonates and children [29]. Therefore, exogenous antioxidants and precursors for the synthesis of antioxidants should be supplied via TPN in neonates.

**Figure 1:**
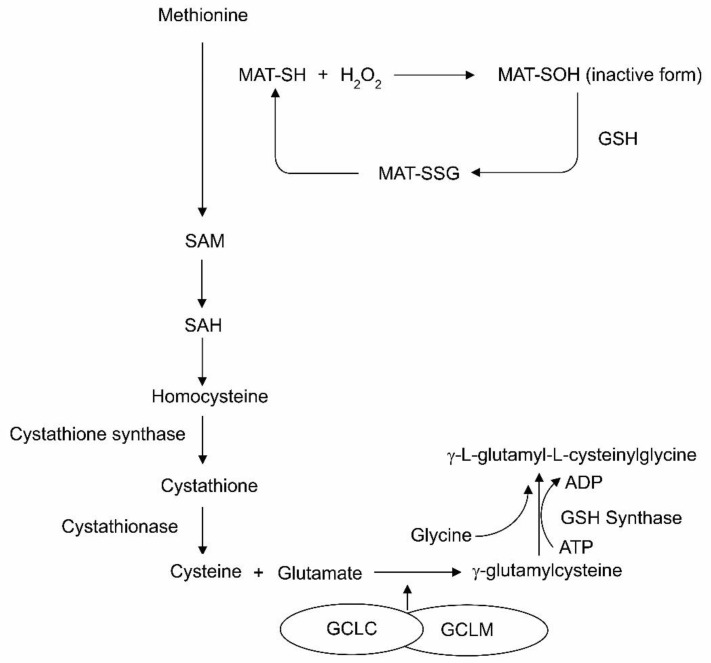
Glutathione biosynthesis and the role of glutathione on the reactivation of MAT-SOH. Hydrogen peroxide (H_2_O_2_) generated in the total parenteral nutrition (TPN) reacts with the –SH group of methionine adenosyltransferase (MAT), converting it to –SOH, which is the oxidized form of the thiol group, resulting in a vicious cycle. The reduced form of glutathione (GSH) reforms the MAT-SH from MAT-SOH by generating MAT–mixed disulfide (MAT-SSG). SAM, S-adenosylmethionine; SAH, S-adenosylhomocysteine; GCLC, catalytic subunit of glutamate–cysteine ligase; GCLM, modifier subunit of glutamate cysteine ligase.

There are also antioxidant systems that depend on the sufficient dietary intake of precursors. For example, synthesis of the most important intracellular antioxidant, glutathione, depends on sufficient dietary cysteine [17]. In order to synthesize glutathione, the active form of methionine adenosyltransferase (MAT-SH), a key metabolic enzyme, involves in transmethylation of the first step of methionine catabolism to provide the precursor, cysteine for the synthesis of glutathione [30]. In the context of TPN, MAT-SH is converted to an inactive form of methionine adenosyltransferase (MAT-SOH) by hydrogen peroxide generated in TPN, resulting in a reduction in the S-adenosylmethionine synthesis [31] (Figure 1). The low activity of MAT-SH is regenerated from MAT-SOH by glutathione. In addition, the detoxification process of peroxides by glutathione peroxidase needs the reduced form of glutathione (GSH) [32]. Most importantly, the availability of cysteine from methionine is a limiting step for the synthesis of glutathione because of the immaturity of cystathionase [17]. Hence, cysteine is deemed a conditionally essential amino acid, and may become essential during TPN feeding. Alternatively, direct glutathione supplementation with at least 10 μM of oxidized glutathione (GSSG) could also have a beneficial effect in lowering the oxidant load [17].

Overall, it is essential to understand the components within TPN can lead to oxidation in vitro as well as in vivo, and how TPN could be adapted to reduce the oxidant load as well as enhance antioxidant capacity in the neonatal body. Moreover, oxidative stress from TPN feeding can come from not only the oxidation of nutrients in vitro in mixed TPN solutions, but also from in vivo reactions from infusing pro-oxidant molecules intravenously, both of which can overwhelm immature antioxidant systems. Hence, this narrative review discusses insights on oxidant molecules generated in TPN, nutrient sources of oxidants, in vivo impacts, and measures to minimize oxidant levels.

To develop this narrative review, we carried out a literature search using primary sources, including original scientific articles and review papers, and secondary sources, including web pages, bibliographic indexes, and databases. PubMed, SciELO, Embase, Scopus, Web of Science, and Google Scholar were used to select records. We used a variety of keywords, including “parenteral nutrition”, “total parenteral nutrition”, “oxidants”, “lipid peroxidation”, “amino acid oxidation”, “parenteral nutrition-associated liver disease”, “gut/intestinal atrophy”, “bronchopulmonary dysplasia”, “micronutrients”, “antioxidants”, and “pro-oxidants”. All articles published up to April 2021 that met the scientific methodological standards related to the main topic and subsections of the review were processed.

## 2. In Vitro Oxidation of TPN

TPN is prepared by combining individual nutrients together into a bag before administration, and is referred to as all-in-one TPN. The interaction among the nutrients within the bag is a major source of oxidants, in part due to chemical reactions among elemental nutrients under various conditions. These chemically altered elemental nutrients in the bag can result in oxidant production that affects metabolism within neonates [5]. For example, the solution can generate oxidants such as hydrogen peroxide and organic peroxides (R-O-O-R’)—compounds possessing one or more oxygen–oxygen bond, as a consequence of redox reactions [33,34,35]. Organic peroxides can be classified into different types of peroxides that correspond to the peroxide structure. Among the classification, hydroperoxides are the major peroxide form; of these, organic (alkyl) hydroperoxides (R-O-OH), are among the more common hydroperoxides. Peroxides further non-specifically react with lipids, amino acids, vitamins, and trace elements in the TPN. For instance, the oxidative degradation of lipids generates lipid hydroperoxides [36], which are the primary products of the free-radical-initiated peroxidation of polyunsaturated fatty acids [37]. These oxidant molecules, together with amino acid oxidation and a high level of elemental pro-oxidant nutrients including vitamin C, copper, and iron within the TPN, may overwhelm neonatal antioxidant systems and cause adverse outcomes in neonates.

### 2.1. Oxidation of Lipid Emulsions for TPN

**Figure 2:**
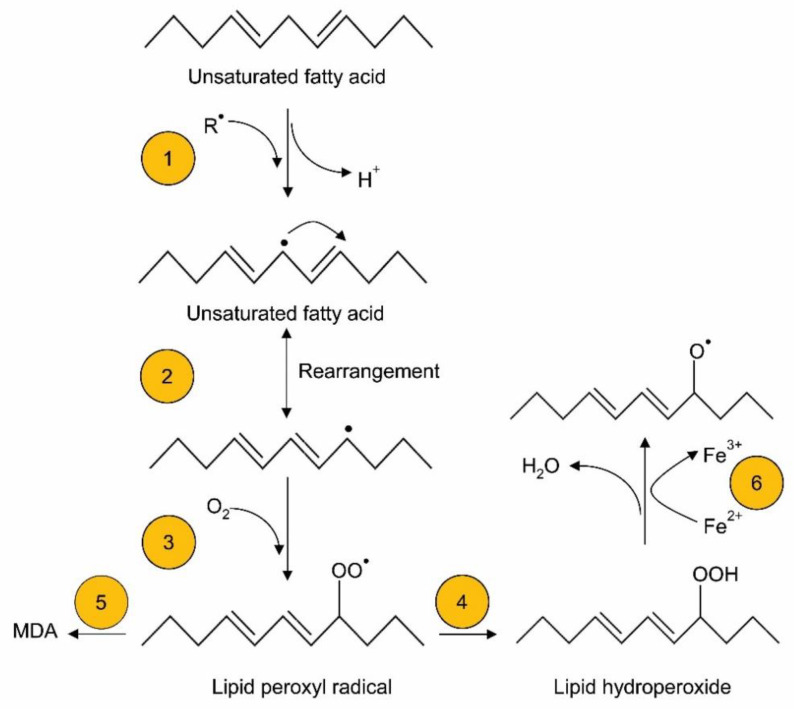
Lipid peroxidation of unsaturated fatty acids. Step 1: abstraction of a proton by free radicals, generating a carbon-centered lipid radical. Step 2: molecular rearrangement to generate a stabilized conjugated diene. Step 3: oxygen reacts with an unsaturated fatty acid radical to form a lipid peroxyl radical. Step 4: the lipid peroxyl radical abstracts H^+^ from another source to generate lipid hydroperoxide. Step 5: the lipid peroxyl radical breaks down to form aldehydes, including malondialdehyde (MDA), 4-hydroxy-2-nonenal (from omega-6 fatty acids) and 4-hydroxy-2-hexanal (from omega-3 fatty acids). Step 6: lipid hydroperoxides can react with Fe^2+^ via Fenton-type reactions, producing LO^•^ radicals.

**Figure 3:**
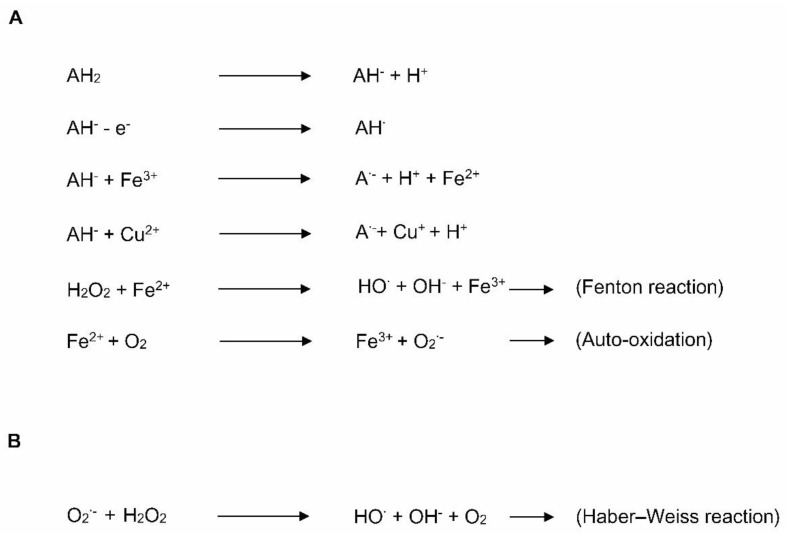
The formation of ascorbate radicals, hydroxyl radicals, and superoxide radicals, generated from ascorbic acid. (**A**) Hydrogen peroxide (H_2_O_2_) reacts with a ferrous ion and generates a hydroxyl radical according to the Fenton reaction; (**B**) the formation of a hydroxyl radical via the Haber–Weiss reaction. AH_2_, ascorbic acid; AH^−^, ascorbate anion; AH^•^, ascorbate radical; A^•−^, dehydroascorbate; O_2_^•−^, superoxide radical; HO^•^, hydroxyl radical.

Lipids are more prone to be oxidized by free radical attack during oxidative stress [37]. Lipids are oxidized by enzymatic and non-enzymatic reactions. Non-enzymatic oxidation is mediated by free radical and non-radical oxidation [38]. In the free radical process, peroxides lead to hydrogen abstraction at sites of unsaturation (or carbon–carbon double bonds) within lipids, resulting in free radical production; the free radicals eventually react with oxygen to generate lipid peroxyl radicals and hydroperoxides [38] (Figure 2). The free radical form, a hydroperoxyl radical formed during lipid peroxidation, plays a crucial role in oxidant injury [36]. Stored lipid emulsions, and especially soybean-based lipid emulsions, which are eventually added to TPN, can generate high concentrations of oxidized lipids even before administration to neonates as part of TPN [39]. Helbock et al. [40] reported that commercial lipid emulsions (such as Intralipid-20%™; rich in omega-6 fatty acids) were contaminated with approximately 300 μM of hydroperoxides. In addition, hydroperoxyl radicals generated in TPN are protonated and generate hydrogen peroxide, which can further react with iron or copper to produce hydroxyl radicals via the Fenton reaction [41,42] (Figure 3A). In Haber–Weiss reactions, superoxide radicals can react with hydrogen peroxide and generate hydroxyl radicals (Figure 3B). Superoxide radicals further react with the oxidized form of metal ions to yield oxygen and reduced forms of metal ions, which can again participate in redox reactions.

A variety of secondary products, including aldehydes, alkanes, alkenes and conjugated dienes, can be formed through secondary reactions during lipid peroxidation reactions [43]. Among the secondary products, 4-hydroxy-2-nonenal is an important oxidative molecule formed from the peroxidation of omega-6-series fatty acids, whereas 4-hydroxy-2-hexenal is generated from the peroxidation of omega-3-series fatty acids. However, the formation of other oxidative products has also been proposed. For example, F_2_-isoprostanes, prostaglandin F_2_-like compounds, are produced by the free-radical-mediated non-enzymatic oxidation of arachidonate [37,44]. With high oxygen tension, the formation of F_2_-isoprostanes is limited [45]. Isofuran, a similar product, containing a substituted tetrahydrofuran ring of F_2_-isoprostanes, has been examined as a marker of oxidative stress during increased oxygen tension. In addition, malondialdehyde (MDA) is another oxidative molecule that can be produced from lipid peroxidation. MDA is highly cytotoxic and can rapidly attach to proteins or nucleic acids in cells [46]. For example, the elevated plasma concentration of MDA in children who received long-term cyclic TPN caused liver damage [47]. Additionally, Weinberger et al. [48] reported that elevated MDA is associated with hepatocellular injury in TPN-administered preterm infants of less than 35 weeks of gestation. MDA detection is used in the thiobarbituric acid reactive substance (TBARS) assay, but it lacks specificity because it measures MDA equivalents [49]; moreover, other aldehydes, including 2-alkenals and 2,4-alkadienals, and additional molecules such as sugars, can react with thiobarbituric acid [50]. Hence, mass spectrometry (GC-MS/MS) methods have been developed to detect MDA, as well as other oxidative markers [51]. Overall, any in vitro assessment of peroxides before the administration of TPN would help to minimize oxidants that are infused into parenterally fed neonates.

The fatty acid composition of different lipid emulsions likely affects the concentration and profile of oxidative products and associated oxidative stress, but few comparative studies exist. In three-day-old guinea pigs, in comparison to Intralipid, a four-day administration of SMOFlipid increased oxidative stress, in terms of increased oxidation of redox potential and apoptosis, and also reduced the alveolarization index [52]. However, the total antioxidant potential was high in infants who received SMOFlipid compared to that in infants who received Intralipid [53]. Although the administration of Omegaven reverses the adverse effects caused by Intralipid and SMOFlipid [54,55], studies on in vitro and in vivo oxidation are limited. Overall, more studies are needed to examine the oxidative status of newer emulsions, such as SMOFlipid and Omegaven, relative to Intralipid.

### 2.2. Oxidation of Amino Acids within TPN

The amino acid formulations for TPN on the market have all nine of the essential amino acids (histidine, isoleucine, leucine, lysine, methionine, phenylalanine, threonine, tryptophan, and valine) and a varying composition of nonessential amino acids, depending on solubility and stability [56]. The composition of amino acids in TPN should be ideal for the rate of protein synthesis and growth of tissues by neonates. Moreover, the composition should also consider that exclusive TPN administration bypasses the gut, which leads to gut atrophy, and this can alter the amino acid requirement profile for neonates [57]. In addition, the amino acid composition should be ideal for other roles beyond protein synthesis, such as nitric oxide (NO) synthesis using arginine; synthesis of the key cellular redox molecule glutathione using glutamate, cysteine, and glycine; inter-conversion of amino acids such as arginine, proline, and glutamate; and methylation reactions using methionine. Thus, the amino acid recommendations for neonates should also accommodate non-protein requirements and be optimum for all functions in a growing neonate.

Despite the importance of the amino acids that are delivered through TPN, several of the amino acids can be oxidized prior to neonatal delivery. Notable amino acids that are quickly modified by oxidation include cysteine and methionine, whereas most other amino acids usually need prolonged exposure to oxidants to become oxidized [58]. For instance, one-electron oxidation of cysteine by reactive oxygen species generates thiyl radicals, whereas two-electron oxidation between cysteine by reactive oxygen species produces sulfenic acid [59,60], which leads to loss of a cysteine possessing protein functions [61]. Although the infusion of high doses of cysteine in TPN to preterm infants is considered safe, it was reported to not increase the amount of plasma glutathione or cystine levels [62], and it increased nitrogen retention in tissues [63]. The other sulfur amino acid, methionine, produces two diastereomer structures—methionine-S-sulfoxide and methionine-R-sulfoxide—during its oxidation, because it contains a prochiral center [64]. Hence, availability of methionine for cysteine and protein synthesis can be limited by oxidation. It is also important to note that cysteine is unstable in TPN solutions, and often undersupplied because of its solubility. However, according to the new guidelines from the American Society for Parenteral and Enteral Nutrition (ASPEN) [65], the supply of L-cysteine as the hydrochloride (HCl) salt to TPN is beneficial, including the acidification of the TPN admixture, which can enhance the solubility of calcium and phosphate.

### 2.3. Role of Vitamins in the Oxidation of TPN Solutions

Premature and low-birth-weight infants require vitamin supplementation to prevent deficiencies because they have low body storage and accelerated growth [66]. Among those vitamins, vitamins E and C are important antioxidants in neonates [67]. Vitamin E is an extracellular lipid-soluble antioxidant, whereas vitamin C is an extracellular water-soluble antioxidant [21,68]. A multivitamin preparation (MVP) that contains all lipophilic and hydrophilic vitamins can be added to TPN to perform the vitamins’ vital functions, including antioxidant activity. However, the anti-peroxide activity of MVP-supplemented TPN or lipid emulsions is still not clear. Lavoie et al. [24] claimed that 6 h of light-protected incubation of a fat-free TPN solution and lipid emulsion (Intralipid—10%) without the admixture of MVP did not significantly generate peroxides, whereas MVP added to a fat-free TPN solution and TPN with a lipid emulsion generated a threefold (from 66 μM to 189 μM) and a twofold (from 126 μM to 244 μM) rise in *tert*-butyl hydroperoxide (TBH) levels, respectively. They also observed that TBH formation further increased to 300 μM when the solutions were exposed to light for six hours [69]. The peroxide activity of MVP can be explained by the presence of polysorbate, riboflavin, and ascorbate.

#### 2.3.1. Polysorbate

Polysorbate is added during the preparation of multivitamin mixtures to solubilize both immiscible lipophilic and hydrophilic vitamins in the same medium. Polysorbate is oxidized when exposed to light, because it contains fatty acid esters of polyoxyethylene sorbitan [70]. Laborie et al. [71] demonstrated that TBH levels slowly increased from 860 μM to 900 μM when TPN was incubated for 24 h with polysorbate (polysorbate 20 (1 mg/L) and polysorbate 80 (1.6 mg/L)). However, the formation of peroxides by polysorbates is quite low compared to peroxides generated by other components in the TPN.

#### 2.3.2. Riboflavin

**Figure 4:**
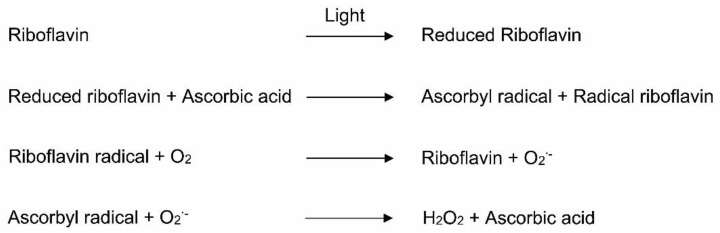
Generation of peroxides in the TPN in the presence of riboflavin and vitamin C. O_2_^•−^, superoxide radical; H_2_O_2_, hydrogen peroxide.

Among the water-soluble vitamins, riboflavin is an important precursor for the synthesis of the biological redox molecules flavin mononucleotide (riboflavin 5′-phosphate) and flavin adenine dinucleotide (adenosine 5′-diphosphate) [72]. The source of riboflavin in MVP is riboflavin 5′-phosphate sodium, which is a highly photosensitive vitamin [71]. Riboflavin in MVP-containing TPN catalyzes the oxidation of ascorbate by oxygen to generate peroxides [73] (Figure 4). After exposure to light, riboflavin undergoes intersystem conversion from a singlet riboflavin to a strongly oxidizing triplet riboflavin state [74]. The triplet riboflavin is then reduced by an electron donor, such as ascorbic acid, to generate an ascorbyl radical and riboflavin radical [73]. The reduced riboflavin reacts with O_2_ and produces superoxide, while regenerating the riboflavin. Superoxide reacts with ascorbyl radicals and hydrogen peroxide [73] (Figure 4). For instance, Kim et al. [73] reported that, after 24 h of incubation, hydroperoxide concentrations were significantly increased in TPN after mixing flavin mononucleotide and ascorbic acid. Photoprotection of riboflavin minimizes the generation of peroxides [73].

#### 2.3.3. Vitamin E

Vitamin E has four molecular structures (α, β, γ, and δ) of tocopherols and tocotrienols. Among the different structures, the α-molecular structure has the highest vitamin E activity. The natural form of the α-tocopherol stereoisomer is the *RRR*-α-tocopherol, whereas all-*rac*-α-tocopheryl acetate is the usual synthetic form. Intralipid predominantly contains the γ-tocopherol form of vitamin E, at 3.8 mg/100 mL; SMOFlipid and Omegaven (rich in omega-3 fatty acids) contain all-*rac*-α-tocopherol, at 20 mg/100 mL and 30 mg/100 mL, respectively. Vitamin E is also known to have distinct biological functions, including the activation of bile acid and xenobiotic metabolism. In addition, tocopherol efficiently prevents lipid peroxidation when another electron donor is present in TPN to reconvert a tocopheryl radical to a non-radical form. Usually, vitamin C acts as an electron donor to the tocopheryl radical to regenerate tocopherol. For instance, clinical and experimental studies have demonstrated that vitamin E supplementation alone does not produce a beneficial effect on oxidative stress leading to atherogenesis, but it works synergistically when co-administered with vitamin C to effectively reduce the oxidative stress [75,76]. For example, Ng et al. [77] found that vitamin E, in Omegaven-supplemented TPN or Intralipid-containing TPN after adding extra vitamin E, prevented the elevation of biliary and lipidemic markers (direct bilirubin, gamma glutamyl transferase, serum triglyceride, low-density lipoprotein, and hepatic triglyceride) of parenteral nutrition-associated liver diseases (PNALD) of preterm piglets. The prevention was believed to be due to the protective mechanism of vitamin E and the presence of other electron donors in the solution. For instance, the inclusion of vitamin E in newer, highly polyunsaturated fish-oil-based emulsions that are available on the market (i.e., Omegaven and SMOFlipid) has been shown to prevent the oxidation of fatty acids [78]. On the other hand, the failure to prevent cholestasis developed by TPN feeding is explained by the presence of high plant sterols and the reaction of α-tocopherol with peroxyl radicals to form α-tocopheroxyl radicals, which leads to further oxidation to α-tocopheryl quinone if lacking electron donors [79,80]. Hence, supplementation of vitamin E and omega-3 fatty acids may protect from oxidative stress.

#### 2.3.4. Vitamin C

The antioxidant activity of vitamin C is supported by several studies [81,82]. Beyond its antioxidant properties, vitamin C also has anti-inflammatory and immune-enhancing functions, and it acts as a cofactor for many enzymes, including hydroxylases and oxygenases [41]. However, humans and only a few other mammals (e.g., primates, bats, and guinea pigs) require L-ascorbic acid in their diet daily, because it cannot be synthesized due to absence of the enzyme L-gulonolactone oxidase [83]. Vitamin C scavenges free radicals, such as hydroxyl radicals, aqueous peroxyl radicals, and superoxide anions, and nonradical species, including singlet oxygen, nitroxide, and peroxynitrite [84]. Vitamin C first releases an electron from ascorbate (AH^−^) to form an ascorbyl radical (A^•−^), and then releases a second electron from A^•−^ to produce the diketone moiety of dehydroascorbic acid. Both the ascorbyl radical and dehydroascorbic acid have low reduction potentials [85]. Hence, these two molecules can neutralize most biologically relevant radicals and oxidants. In addition, Buettner and Jurkiewicz [86] reported that the ascorbyl radical has minimal reactivity because of its resonance stabilization of the unpaired electron (k_2_ = 2 × 10^5^ M^−1^s^−1^). Dehydroascorbic acid serves as the strong reducing form of ascorbic acid and defends it from oxidation. In addition to the scavenging action of vitamin C, it regenerates other antioxidants, including α-tocopherol and glutathione [87]. The advantage of vitamin C is that it can be regenerated from the ascorbyl radical and dehydroascorbic acid by enzymatic and non-enzymatic pathways [84].

In contrast to the above beneficial effects, high doses of vitamin C could exhibit pro-oxidant effects because the hydroxyl groups of ascorbic acid are reactive, towards singlet oxygen, hydroxyl radicals, hydroperoxide radicals, and hydrogen peroxide. For example, hydrogen peroxide generated in TPN solutions reacts with dehydroascorbate spontaneously to produce ascorbylperoxide [88,89], which is associated with detrimental health outcomes. In the presence of iron, the ascorbate could reduce Fe^3+^ to Fe^2+^, subsequently resulting in the generation of an ascorbate radical [86] (Figure 3A). In addition, the electrons from ascorbate can reduce Cu^2+^ to Cu^+^, and eventually generate superoxide as per Haber–Weiss reactions, as mentioned above. In newborn guinea pigs, ascorbylperoxide causes hypo-alveolarization and the apoptosis of lung tissue as well as higher glutathione redox potential with increasing ascorbylperoxide concentration [18]. Mohamed et al. [90] observed higher urinary ascorbylperoxide levels in infants who were TPN-fed for seven days.

The stability of ascorbic acid in TPN is also another reason for its efficient protection from oxidants. For example, ascorbic acid is more stable in an acidic pH. The stability of ascorbic acid is altered when the solution is exposed to oxygen, light, high temperature, and pro-oxidants such as iron and copper. Burge et al. [91] reported that vitamin C measured by the spectrophotometric method of dinitrophenylhydrazine in a TPN solution containing 10% amino acids, 50% dextrose, and multivitamin (100 mg ascorbic acid) was relatively stable for the first eight hours, and after that, the vitamin C level dropped gradually, such that by 32 h, the concentration of ascorbic acid decreased to 74% of the original concentration. Burge et al. [91] also claimed that the loss of ascorbic acid was higher (60% of the original concentration) when the added trace element solution contained 1.2 mg copper sulfate, compared to TPN without copper. The pro-oxidant activity of vitamin C can be avoided by maintaining an optimum concentration of vitamin C and providing other electron donors.

### 2.4. Role of Trace Elements in Oxidation of TPN Solutions

Trace elements are vital micronutrients in TPN. They play an important role in physiological and metabolic functions, including enzymatic reactions. Substantial studies on trace elements in TPN solutions have focused on the prevention of micronutrient deficiencies [92,93,94], although toxicity is also a concern with the intravenous infusion of minerals, which bypasses key excretory regulation mechanisms for many minerals, especially iron, copper, and zinc [95]. However, studies evaluating the oxidative effect of trace elements in TPN are limited. Steger and Mühlebach [96] reported that peroxide levels (as a relative peroxide value) were significantly increased from 0.52 to 1.92 when mixing the trace elements (2.79 mg iron, 3.27 mg zinc, 0.27 mg manganese, 0.32 mg copper, 0.026 mg chromium, 0.024 mg selenium, 0.019 mg molybdenum, 0.95 mg fluoride, and 0.13 mg iodine per 10 mL) with TPN and incubating the mixture at room temperature for 19 days. These higher peroxide levels could be explained by the presence of pro-oxidants such as iron, copper, manganese, and zinc.

Intravenous administration of iron has been proposed in preterm neonates because the intestinal absorption of iron is poor during the first weeks of life [97,98]; however, caution is warranted. Free iron stimulates the formation of free radicals, whereas conjugated iron (e.g., iron dextran, iron gluconate) prevents the formation of peroxides in TPN solutions. Conversely, Grand et al. [99] used iron saccharate in TPN, and they reported that the lipid peroxide levels (as MDA) increased from 810 nM to 1586 nM within a 24-h incubation without light exposure. This is supported by Grand et al. [99], who showed that the concentration of MDA in an all-in-one TPN solution with iron was high, suggesting that the generation of lipid peroxides formed quickly when iron was present in TPN solutions. Another aspect to consider is that prolonged iron infusion via TPN can lead to dangerously high iron levels, because the body does not have an effective excretory system for absorbed iron [95]; this high iron status could prolong oxidative damage well beyond TPN feeding.

Iron and copper in TPN can also further catalyze oxidative reactions through interactions with other nutrients. For instance, ascorbic acid readily induces hydrogen peroxide generation in the presence of Cu^2+^ [100]. Although iron is a highly reactive metal, as well as being a strong biological oxidant and a reducing agent, the pro-oxidant activity of copper is more pronounced than iron [100]. Zinc and manganese may have similar effects on the generation of peroxides, but less is known about those interactions.

## 3. Parenteral Nutrition and Environmental Conditions

In addition to the nutrient composition of TPN, its preparation and storage environment can also contribute to the oxidation of its components. As briefly discussed above, light exposure is a key factor that can increase oxidant levels, but TPN can also generate additional oxidants when exposed to oxygen during preparation, storage, and infusion at the bedside. Moreover, the temperature in neonatal intensive care units can induce the formation of oxidative molecules. It is essential to minimize these conditions to limit the production of oxidants in TPN.

### 3.1. TPN Exposure to Light

As briefly mentioned, the exposure of TPN to ambient light or to light during the phototherapy of a neonate induces the generation of peroxides. Indeed, the oxidation of lipid emulsions is particularly immense when exposed to ambient light or phototherapy in a clinical setting. For example, Laborie et al. [101] found that the peroxide concentration in light-exposed TPN was between 190 and 300 μM, compared to 60 and 130 μM when TPN was protected from light. Peroxides generated in vitro from light exposure including 4-hydroxy-2-nonenal, MDA [99,102] and 4-hydroxy-2-hexenal have been identified in lipid emulsions [33]. Moreover, many light-induced reactive oxidative species can interfere with endogenous NO levels, resulting in increased vasoconstriction and exacerbating physiological effects of TPN feeding.

Fat-free TPN, including amino acids and vitamin mixtures, can also be contaminated with hydrogen peroxide after mixing with light-exposed riboflavin [103,104]. In another study, a light-exposed mixture containing 10% dextrose, amino acids, and electrolytes generated 25 μM peroxides, but photo-protection over six hours at room temperature did not result in peroxide production [5]. Interestingly, the concentration of peroxides jumped threefold (75 μM) after adding a lipid emulsion. Notably, the addition of 1% MVP induced the generation of peroxides to 350 μM, even after two hours of incubation; however, the concentration of peroxides dropped to 250 μM when protecting the solution from light [5]. Therefore, the generation of peroxides in TPN is dependent on numerous combinations of nutrients, but light protection is critical to minimize many of these interactions.

The protection of TPN from photo-oxidation in a clinical setting is challenging to achieve [24] because it is difficult to protect the TPN bag and its connected tubing efficiently. However, some studies have demonstrated effective photo-protection by covering it with aluminum foil or opaque plastic polythene. For instance, Laborie et al. [71] examined peroxide levels in a TPN bag which was shielded from light using a black garbage bag, and by using different coloured tubing including orange, yellow, and black. They observed that yellow tubing was as effective as a completely opaque black tube or tube covered with aluminum foil in preventing further oxidation when exposed to light. Indeed, the yellow tube was more suitable to see air bubbles or precipitation than the black tube [71]. Moreover, protection from light during product preparation must also be considered. The generation of free radicals in any solution can be lowered simply by shielding TPN from light during preparation [105], although how practical that is in a manufacturing plant or hospital pharmacy needs to be evaluated.

### 3.2. TPN Exposure to Oxygen

A TPN solution exposed to oxygen during its preparation and infusion to a neonate gains oxidant molecules at each step. Oxygen is the basis of aerobic life of living organisms, but it is frequently reactive by itself [106]. It reacts with an electron from a donor, such as a monounsaturated or polyunsaturated fatty acid, certain amino acid residues such as tyrosinyl- or tryptophanyl-residues, or vitamin C, to generate a superoxide anion. Laborie et al. [101] demonstrated that the removal of oxygen by nitrogen from a test solution of neonatal TPN inhibited the generation of peroxides, but they also observed that the effect of the oxygen washout was lost when the solution was subsequently infused via an intravenous infusion set. The removal of oxygen from preparation to infusion would be a difficult task. For example, even if all the air was removed from TPN bags, the effect to minimize the peroxides is lost because the chance of oxygenation is high when administering the TPN to neonates [19].

### 3.3. Effect of Storage on Oxidants in TPN Solutions

Within TPN, amino acids are typically stable for about four months at 2–8 °C, except for cysteine, which slowly dimerizes to yield cystine, giving a yellow discoloration of parenteral solutions with storage time [107]. Pitkänen et al. [108] reported that lipid degradation and the production of oxidants were high during the storage of Intralipid. They determined that levels of pentane, generated from the peroxidation of omega-6 fatty acids [109], were significantly higher after six months of storage after adding Intralipid to phosphate-buffered saline [108]. They also found that the infusion of lipid emulsions increased the concentration of exhaled pentane during the first week of life in premature infants, suggesting the significant peroxidation of lipids in TPN during storage. Intravenous infusion of TPN contaminated with oxidants in vitro can overwhelm the antioxidant capacity of neonates and cause adverse effects.

On the other hand, the materials used to manufacture TPN bags may also affect oxidation reactions and lead to harmful effects in neonates. For instance, until recently, most TPN bags were made of polyvinyl chloride (PVC) with flexible plasticizers of di(2-ethylhexyl) phthalate (DEHP) [110], which is a carcinogenic, hepatotoxic, and teratogenic chemical compound [111]. Lipid-soluble DEHP is leached from PVC when mixing with lipids [111], which could result in increased tissue uptake. Studies have reported that detectable amounts of DEHP in intravenous solutions stored in PVC bags were indeed observed [110,111,112]. An alternate material, ethylvinyl acetate (EVA), which exhibits good flexibility and high resistance to external influences and adhesion, has also been used in TPN bags [113]. However, Balet et al. [114] found that hydroperoxides produced in TPN mixtures that were stored within multilayer bags containing both EVA and polyvinylidine were lower than in the TPN mixtures that were stored in EVA-only bags for 14 days at 37 °C. However, the MDA generated in the two bags was not significantly different.

In addition to the TPN bags, the composition of medical tubing also needs to be considered. The tube used between the TPN bag and a venous catheter is made of PVC–DEHP. Loff et al. [115] reported that before starting a perfusion of TPN using the catheter line, the DEHP concentration was nearly 0.06 μg/mL. After perfusion, the concentration of DEHP in the solution jumped to approximately 2 μg/mL. This is consistent with other recent studies [116,117]. Hence, alternative plasticizer-free materials need to be investigated.

## 4. In Vivo Impact of Oxidized TPN after Infusion

Although TPN is a life-saving therapy for sick neonates, it is linked to harmful complications after prolonged administration [12]. Numerous in vivo studies have been carried out to examine the effects of oxidant molecules from contaminated TPN after infusion. Clinical studies show that additive effects of O_2_ supplementation and TPN administration caused oxidative stress and an increased risk of bronchopulmonary dysplasia (BPD) in neonates under 29 weeks of gestation [118]. Plasma F_2_-isoprostane levels were increased in preterm neonates between 23 and 28 weeks of gestation, who received olive-oil- or soybean-oil-based lipid emulsions [119]. In an observational study, Unal et al. [120] identified oxidative stress after a seven-day infusion of either SMOFlipid or ClinOleic (a mixture of refined soybean oil 20% and refined olive oil 80%) in very-low-birth-weight infants between 25 and 32 weeks of gestation. Additional clinical studies are needed to examine metabolic changes in response to oxidized TPN, as well as tissue-specific changes to the human metabolome and proteome. In animal models, Chessex et al. [121] reported that light-exposed MVP-supplemented TPN was associated with an elevated urinary excretion of nitrogen (by 40%) compared to control TPN in three-day-old guinea pig pups. Another study reported that guinea pigs on light-exposed TPN had more hepatic steatosis, higher liver weight, and elevated isoprostane F_2α_ concentrations compared to animals fed light-protected TPN [19,122]. As already noted for clinical studies, detailed animal studies are needed to examine metabolic complications, including glucose intolerance, which may arise due to the extensive use of TPN in neonates [12]. However, the most established complications due to prolonged TPN feeding are PNALD, gut atrophy, and BPD.

### 4.1. Parenteral Nutrition, Oxidant Load and Liver Diseases

PNALD is defined as a heterogeneous injury of the liver, characterized by cholestasis, steatosis, and, eventually, fibrosis and cirrhosis [77,123]. Several animal experiments in piglet, guinea pig, and rat models have demonstrated that a continuous infusion of oxidized TPN solution for some days affects hepatobiliary function. Bhatia et al. [124] claimed that 10 days of infusion of light-exposed TPN to rats leads to hepatobiliary disease. Morin et al. [17] also observed liver steatosis after 5 days of TPN in one-month-old guinea pigs. TPN feeding for 7 days to male one-month-old Sprague Dawley rats also led to the development of liver diseases [15]. Additionally, in humans, newborn infants (>1000 g) who received TPN for a longer period (>7 days) developed PNALD [14]. Although TPN-induced PNALD is a well-established consequence of intravenous feeding, its aetiology is still unclear.

The reasons for the development of PNALD in neonates may be due to the composition of omega-3 and omega-6 fatty acids, the amount of phytosterols, parenteral lipid load, and different compositions of non-lipid nutrients in TPN [78,125,126]. For example, soy-based parenteral lipid emulsions, containing high concentrations of phytosterol and omega-6 fatty acids, have become known as risk factors for cholestasis and hepatocellular damage [79]. This association manifests as a significant association between the accumulation of circulating phytosterols and the elevation of liver enzymes in neonates [127]. The accumulation of phytosterols also leads to higher bile acid secretion and causes cholestasis in TPN-fed piglets [77]. In fact, long-chain polyunsaturated fatty acids are more prone to damage by peroxidation, resulting in free radical peroxide production, which can contribute to the liver injury detected in PNALD [125,128]. Lipid emulsions with vitamin E undoubtedly reduce the risk of peroxidation due to their antioxidant capacity, and protect lipid membranes from oxidation [129]. Omegaven, a lipid emulsion containing purely fish oil, also has hepatoprotective effects [130,131], which may be due to its rich content in vitamin E and/or the lack of phytosterols. In addition to the above number of aetiologies hypothesized to explain PNALD, the lack of enteral feeding is also one of the key reasons for PNALD, because it leads to gut atrophy and disruption of the enterohepatic circulation of bile acids [16,132]. Hence, liver disease may also be due to intestinal failure, as a result of exclusive PN feeding, which is referred to as intestinal failure-associated liver disease [133,134]. The development of PNALD in neonates is multifactorial; therefore, the term “intestinal failure-associated liver disease” is preferred to explain liver disease due specifically to intestinal failure as a result of TPN feeding [135].

The absence of enteral feeding prevents the stimulation of receptors, hormones, and growth factors. It also blocks the normal gut–liver crosstalk by reducing downstream signaling to the liver via portal circulation [136]. For example, farnesoid X receptor (FXR), a ligand-activated transcription factor, is expressed in the terminal ileum and is regulated by bile acids. Lack of enterohepatic circulation suppresses the FXR and reduces hepatic bile acid production by modulating cholesterol 7-α-hydroxylase (CYP7A1). Reduced FXR expression subsequently reduces the activation of fibroblast growth factor 19 (FGF19), which reduces protein synthesis in the liver and may exacerbate liver injury due to PNALD [137]. Bile acid absorption in the ileum during enterohepatic circulation is linked with the stimulation of FXR [138]. Thus, the enteral administration of chenodeoxycholic acid can serve as a ligand for FXR, thereby preventing hepatic injury [132]. The gut microbiota also performs a vital function in the health of infants. It has been suggested that the administration of TPN alters the composition of gut microbiota because of the lack of enteral feeding and starvation of bacteria, which leads to bacterial translocation. Exclusive TPN can also cause the favourable growth of Gram-negative, endotoxin-producing bacteria, which can exacerbate systemic bacterial infection. These events can result in the suppression of bile acid transporters, and eventually, hepatic injury by endotoxin- and cytokine-mediated suppression [136]. Although the mechanisms are complex, the lack of enteral stimulation during TPN feeding can lead to the impaired enterohepatic metabolism of bile acids, leading to profound liver injury and potentially life-threatening sepsis.

### 4.2. Parenteral Nutrition and Gut Atrophy

In growing neonates, exclusive TPN leads to significant functional and morphological gut atrophy; however, the mechanisms and consequences of atrophy are poorly understood. The atrophied gut leads to a reduction in intestinal metabolic capacity, diminished absorptive capacity, and compromised de novo synthesis of many nutrients, including polyamines and amino acids such as arginine [139]. Moreover, prolonged gut atrophy also leads to intolerance to the reintroduction of oral feeds and complicates the transition from parenteral to enteral feeding. Niinikoski et al. [140] reported that gut atrophy is a direct result of a parenteral feeding-induced rapid suppression of blood flow in the superior mesenteric artery (by 30% in under eight hours), which preceded small intestinal tissue atrophy and lowered protein synthesis. As a result, clinical practice during PN feeding often includes minimal enteral nutrition, which involves small volumes of oral feeding to stimulate gastrointestinal function and growth to prevent atrophy. The mechanism by which enteral stimulation improves enteral feeding tolerance is still unclear, but the primary physiological outcome necessary for improving gut atrophy and recovery during TPN is improved mesenteric blood flow [140]. Previously reported studies have shown that mesenteric blood flow can predict the early feeding tolerance of preterm infants [141,142]. Notably, small-intestinal blood flow is regulated by NO, which is synthesized from arginine. In neonates, because arginine synthesis depends on small-intestinal metabolism [139], gut atrophy exacerbates arginine availability and NO synthesis, further reducing intestinal blood flow. Hence, arginine-supplemented TPN can help maintain the integrity of the small intestine through an increased rate of protein synthesis and migration of enterocytes and serves as a precursor of NO synthesis [143].

As already discussed, NO availability is also sensitive to oxidative stress. Huber et al. [144] conducted a study to examine the effects of light-protection of TPN and of N-acetyl cysteine (NAC), the limiting amino acid for the synthesis of glutathione (i.e., the primary intracellular antioxidant), on the superior mesenteric artery blood flow, gut morphology, and oxidative status of piglets. They found that the superior mesenteric artery blood flow rate declined over six days for all treatment groups (light-protected TPN, light-protected NAC-enriched TPN, light-exposed TPN, and light-exposed NAC-enriched TPN), consistent with previously observed effects of TPN [140]. However, by day 6 of TPN feeding, the light-protected TPN group showed only a 34% reduction in blood flow from baseline, which was significantly better than the 45% to 63% reduction in blood flow in the other groups. They concluded that the photoprotection of the TPN solution effectively ameliorated the PN-associated decline in the superior mesenteric artery blood flow. However, NAC supplementation surprisingly offset this amelioration. They also observed a 25% reduction in hepatic lipid peroxidation when TPN was protected from light. Therefore, TPN redox status can affect functional outcomes in the neonatal gut, and minimizing in vitro oxidation in TPN would have clinical impacts.

The mechanisms behind the lack of enteral stimulation and gut atrophy may also involve enterohepatic pathways. For example, some animal studies have shown that the protein-coupled bile acid-activated receptor, also called Takeda G protein-coupled receptor (TGR5), rich in the crypts of the intestine, is involved in the regulation of gut atrophy [145,146,147]. The expression of TGR5 is regulated by primary and secondary bile acids [148]. Jain et al. [149] observed that the administration of an agonist of TGR5, namely, oleanolic acid, which stimulates TGR5 expression, reduces villous atrophy by increasing the villous height/crypt depth ratio of TPN-fed piglets, and doubling small intestinal weight. Guzman et al. [16] also reported that TPN feeding to piglets for 14 days resulted in a significant elevation of serum bilirubin (a biomarker of cholestatic liver injury), serum bile acids, bile acid deposition within intra-parenchymal cells, and an increased hepatic cholestasis score, compared to enterally fed piglets. They also showed significant villous atrophy and reduction in the thickness of muscularis mucosa with TPN feeding. Molecular studies on gut-systemic signaling regulators revealed that TPN-fed piglets exhibited a downregulation of liver FXR expression, liver constitutive androstane receptor (CAR), gut FXR, G-coupled bile acid receptor, epidermal growth factor (EGF), organic anion transporter (OAT), mitogen-activated protein kinase-1 (MAPK1), and sodium glucose-linked transporter (SGLT-1), compared to enterally fed piglets [16]. These studies together suggest that gut atrophy could be due to reduced blood supply as well as an interruption of hepatobiliary circulation.

### 4.3. Parenteral Nutrition and BPD

BPD is a chronic pulmonary disease of preterm neonates. The aetiology of BPD is multifactorial, including ventilatory injury, prenatal inflammation and infection, and hyperoxia. Oxidative stress is one of the important factors causing BPD in neonates [90]. Infants who excreted higher concentrations of urinary ascorbyl peroxides were significantly more likely to develop BPD [90]. A guinea pig model demonstrated that the oxidant molecules in TPN cause adverse effects on biochemical and histological parameters in the lungs [18]. Indeed, low-birth-weight infants fed TPN contaminated with ascorbyl peroxides were more likely to develop BPD [90]. Lavoie et al. [150] reported that the peroxides from TPN caused increased collagen deposition in the alveoli and increased the gene expression of procollagen mRNA. Animals infused with MVP or TPN had reduced alveolarization, even when these solutions were adequately photo-protected [151]. Adding the antioxidant glutathione to TPN may have beneficial effects on lung health via the reduction in apoptosis, maintenance of redox potential, and elevation of the alveolarization index of lung tissue [17,152]. From these animal and clinical studies, it is clear that oxidant load is a key predictor of lung function in rapidly developing neonates.

### 4.4. Biomolecules in the Body Affected by Oxidized TPN

Generally, outcomes of oxidative stress occur as a result of an imbalance between pro-oxidant and antioxidant levels. An infusion of hydroxyl radicals and hydroperoxyl radical-contaminated TPN attacks cell membrane lipids. Indeed, reactive hydroperoxides further react with polyunsaturated fatty acids in cell membranes and produce lipid peroxyl radicals, and eventually lipid peroxides, through three steps: initiation, propagation, and termination [36] (Figure 2). Brain tissue is extremely susceptible to oxidative injury because it consumes a relatively high amount of oxygen compared to other tissues [153], and it has an abundant amount of polyunsaturated fatty acids. For instance, neuroprostane, which is produced from the free-radical-mediated oxidation of docosahexaenoic acid in nervous tissue, is highly concentrated in the neuronal membrane. Cholesterol and linoleate are abundant lipids in vivo, and their free-radical-mediated oxidations yield 7-hydroperoxycholesterol (7-OOHCh) and hydroperoxyl octadecadienoate (HPODE), respectively. The aldehydes in TPN, including 4-hydroxy-2-nonenal, are highly reactive, and they damage proteins and DNA to produce carbonylated proteins and 7,8-hydroxy-2′-deoxyguanosine, respectively [154]. Similarly, oxidative lipid products in circulation can cause the oxidation of low-density lipoproteins, which ultimately leads to cardiovascular diseases. Research on oxidized lipoprotein levels in infants receiving TPN are limited, but likely play a key role in metabolic perturbations, which may have long-term consequences.

## 5. Conclusions

Many of the clinical complications in TPN-fed neonates can be related to oxidative stress in early life. Newborns have an immature and inadequate antioxidant system to neutralize the oxidants generated in TPN, and administered via intravenous feeding regimens. Moreover, typical TPN solutions contain high concentrations of pro-oxidant nutrients, with few antioxidants that could limit oxidant formation. Furthermore, typical environmental conditions can induce the formation of oxidants in TPN, including light exposure and/or clinical phototherapy while receiving TPN, exposure to oxygen during the preparation and infusion of TPN, and inadequate long-term storage conditions leading to the instability of nutrients. Major peroxides found in TPN are hydroxyl and hydroperoxyl radicals, MDA, isoprostane and 4-hydroxy-2-nonenal. Infants exposed to contaminated TPN for an extended period of time may develop clinical complications, including PNALD, BPD, hepatobiliary dysfunction, and gut atrophy. Moreover, the oxidation of amino acids limits some essential amino acids for vital functions, including NO synthesis, which is required to maintain blood flow to compromised organs. Evidence from various animal and clinical studies recommends the supplementation of antioxidants, and the reduction in pro-oxidants in TPN, as well as the optimization of environmental conditions to limit oxidant formation during TPN manufacture and delivery. These modifications also need to consider the dietary adequacy of nutrients for TPN-fed neonates. The evidence suggests that some potentially beneficial modifications include higher arginine and cysteine for NO and glutathione synthesis, respectively, reductions in iron and copper concentrations, and protection from light; such modifications to optimize TPN to minimize oxidant load also need to be systematically tested in a clinical setting.

## Data Availability

Not applicable.
